# Definition and characteristics of acute exacerbation in adult patients with chronic rhinosinusitis: a systematic review

**DOI:** 10.1186/s40463-020-00459-w

**Published:** 2020-08-18

**Authors:** Dawei Wu, Benjamin Bleier, Yongxiang Wei

**Affiliations:** 1 Department of Otolaryngology-Head and Neck Surgery, Beijing Anzhen Hospital, Capital Medical University, Anzhen Road 2, Chaoyang District, Beijing, 100029 China; 2 Department of Otolaryngology, Massachusetts Eye and Ear Infirmary, Harvard Medical School, 243 Charles Street, Boston, MA 02114 USA

**Keywords:** Chronic rhinosinusitis, Nasal polyps, Acute exacerbation, Definition, Clinical characteristics, Immunologic characteristics, Risk factors, Etiology

## Abstract

**Background:**

Acute exacerbations (AE) in chronic rhinosinusitis (CRS) have been increasingly recognized as an important clinical issue. The purpose of this study is to summarize the current definitions and evaluation parameters of AE and then identify and quantify the clinical and immunopathologic characteristics of AE in CRS.

**Methods:**

A systematic review of the literature was performed on PubMed, Scopus, and Cochrane databases from January 1990 through August 2020 to identify studies relating to AE in CRS. Exclusion criteria included non-English and non-human studies, and case reports.

**Results:**

The definitions of AE in CRS among all the studies were based on a description of short-term worsening sinonasal symptoms. Patient-reported sinus infection and exacerbation related medical treatment during the preceding 3 months to 1 year were used to evaluate the frequency of AE in CRS. The average decline in 22-item Sino-Nasal Outcome Test (SNOT-22) score during an exacerbation was 7.83 points relative to baseline. Comorbid asthma, SNOT-22 scores ≥24, allergic rhinitis, eosinophil count ≥150/μL and autoimmune disease were positively associated with an exacerbation-prone CRS phenotype. AE in chronic rhinosinusitis with nasal polyps (CRSwNP) was associated with increased expression of mucus cytokines including myeloperoxidase (percentage increase [PI] = 101%), IL-5 (PI = 125%), and IL-6 (PI = 162%) and could be predicted by the increasing mucus cystatin and periostin.

**Conclusion:**

The definition of AE in CRS is largely driven by patient-reported symptoms and is associated with several risk factors. Quantitative changes in mucus cytokines associated with AE in CRSwNP and may be used to predict events. The development of a consistent definition of AE in CRS is critical to help define disease control and treatment efficacy.

## Introduction

Chronic rhinosinusitis (CRS) is often associated with a fluctuating disease course. Acute exacerbations (AE) in CRS have been shown to directly account for increasing healthcare costs [1, 2], annual physician visits [3], and significant decreases in workplace productivity [4, 5]. Recently, the frequency of AE has been identified as an independent predictor of quality of life [6]. Based on the current guidelines, an acute exacerbation is defined as an acute and transient worsening of preexisting symptoms in patients with CRS [7, 8]. However, there is no consensus definition of how to quantify AE due to multifactorial etiologies and inconsistency in endpoint reporting.

Prior attempts to report on AE have relied on empirical clinical criteria. For example, Rank et al. utilized diagnosis coding and at least one of the following: prescription for systemic antibiotics, systemic corticosteroids, plans for surgical intervention, emergency department or urgent care visit, or hospitalization for CRS [9]. Similarly, Sedaghat et al. used three metrics to assess the frequency of AE including patient-reported sinus infections, CRS-related antibiotic courses, and CRS-related oral corticosteroid courses, each over the preceding 3 months [6, 10, 11]. These direct treatment-related metrics of AE facilitate assessment on the AE in CRS but fail to correlate the AE in CRS with both degree of subjective changes in clinical symptoms and objective measures of inflammation.

The purpose of this study is to therefore utilize a systematic review of the literature to summarize the current definitions and evaluation parameters of AE and then quantify both the clinical and immunopathologic characteristics of AE in patients with CRS.

## Methods

An evidence-based systematic review was performed utilizing the Preferred Reporting Items for Systematic Reviews and Meta-Analysis (PRISMA) guidelines. A comprehensive search of PubMed, Scopus, and Cochrane databases from January 1990 through August 2020 was conducted to identify studies relating to the AE in CRS. A combination of terms was used to maximize the probability of finding all relevant publications: acute exacerbation, exacerbation, chronic rhinosinusitis, rhinosinusitis, chronic sinusitis, nasal polyps and polyposis.

### Study selection

**Fig. 1:**
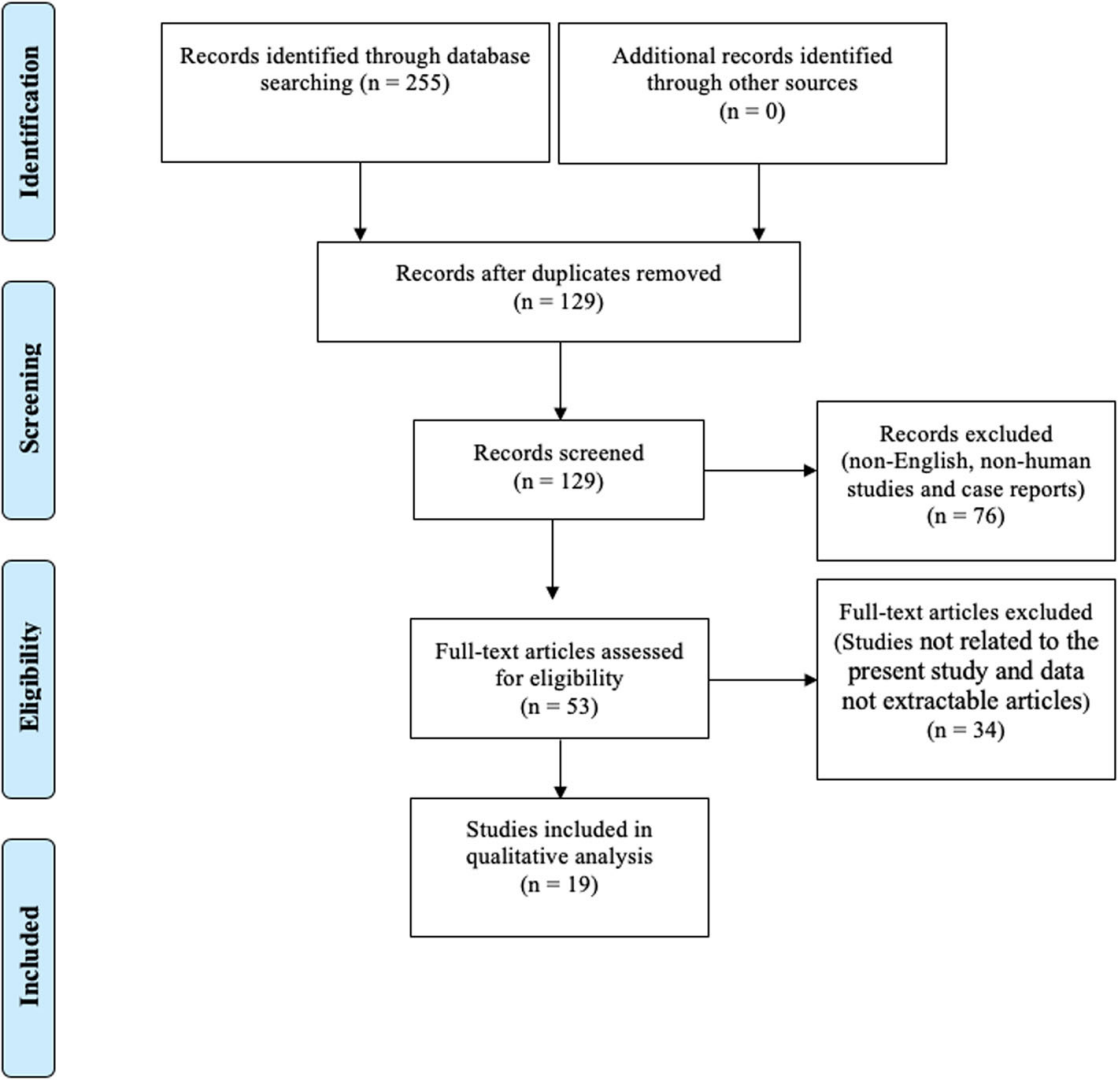
Flow diagram of the study

Titles and abstracts of all the relevant studies were reviewed by 2 independent authors (DW and YW). Included studies addressed the etiology, characteristics, and diagnosis of AE in CRS. All included studies were downloaded and the full-text was reviewed by both authors. Studies were excluded if they were: non-English, non-human studies, case reports, or not related to the present study. Figure 1 outlines the search strategy and inclusion process used to find relevant studies.

### Data extraction and analysis

Data included year of publication, study design, age range, diagnostic criteria, bacterial findings, immunohistologic findings, risk factors, and conclusions. After analysis of each article, summary tables were developed. The quality of included studies was determined from the Oxford Center for Evidence Based Medicine Levels of Evidence categorization, based on study design as well as clinical endpoints [12].

## Results

### Included studies

The initial database search identified 255 articles (Fig. 1). Duplicates, non-full text, non-English, and data not extractable articles were excluded (*n* = 239). A total of 19 articles underwent a full-text assessment for eligibility and met the final inclusion criteria for systematic review. Nine studies explored the clinical characteristics of AE in CRS including the clinical metrics and risk factors of AE in CRS. Another seven articles focused on the in vivo immunologic characteristics associated with AE in CRSwNP.

### Diagnosis of AE in adult patients with CRS

Diagnostic criteria regarding the presence of AE in CRS were summarized (Table 1). The definitions of AE in CRS among all the studies were based on subjective descriptive changes in sinonasal symptoms. These included a sudden worsening of CRS symptoms with a return to baseline symptoms following treatment. Medical treatments during AE in CRS included short-term administration of antibiotics and corticosteroids.

**Table 1:** Descriptive diagnosis of acute exacerbations in adult patients with CRS

Author/year	Definition of an acute exacerbation in CRS
Zemke, et al. (2019) [13]	The presence of increased nasal congestion, and facial pain; increased sinonasal discharge; usually presence of an unscheduled sick visit.
Orlandi, RR, et al. (2018) [8]	AE in CRS is defined in a patient in whom a previous diagnosis of CRS exists, and a sudden worsening of symptoms occurs, with a return to baseline symptoms following treatment.
Divekar, et al. (2015) [14]	A natural exacerbation was defined as patient-reported worsening of sinonasal symptoms (i.e. runny nose, nasal congestion, and nasal obstruction).
Fokkens, et al. (2012) [7]	A history of sudden worsening of preexisting symptoms suggests an acute exacerbation of chronic rhinosinusitis, which should be diagnosed by similar criteria and treated in a similar way to ARS.
Kuiper, et al. (2018) [15]	Self-reported medication use (antibiotics and oral corticosteroids) for worsened nasal and sinus symptoms; self-reported worsened purulence in the past 4 weeks.
Rank, et al. (2010) [16]	Systemic antibiotics; systemic corticosteroid; plans for a semi-urgent surgical intervention; emergency department or urgent care visit, or a hospitalization for CRS.
Reh, et al. (2009) [17]	Worse nasal symptoms

*CRS* Chronic rhinosinusitis, *CF* Cystic fibrosis, *AE* Acute exacerbation, *ICAR: RS* International Consensus Statement on Allergy and Rhinology: Rhinosinusitis, *EPOS* European position paper on rhinosinusitis and nasal polyps, *ARS* Acute rhinosinusitis

### Quantitative clinical metrics of AE in CRS

**Fig. 2:**
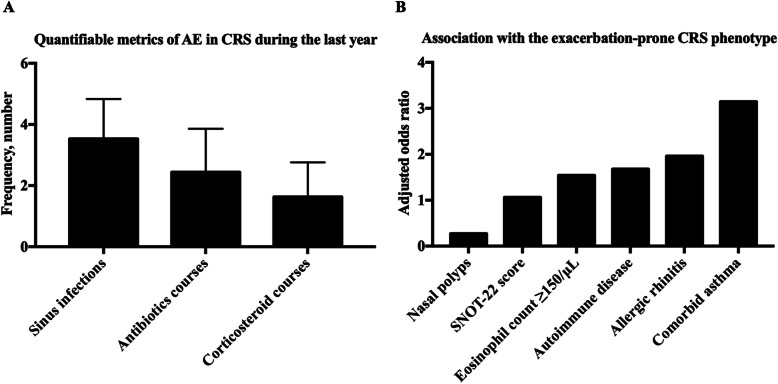
Clinical characteristics of acute exacerbations in adult patients with CRS. **a**, quantifiable metrics of AE during the last year. **b**, association with the exacerbation-prone CRS phenotype. AE, acute exacerbation; SNOT-22, 22-itemSino-Nasal Outcome Test

The evaluation parameters of AE in CRS in seven studies were summarized (Table 2 and Fig. 2). Number of sinus infections, CRS-related antibiotic use, and CRS-related oral corticosteroid use over prior time period were utilized in five studies to evaluate the frequency of AE in CRS [6, 11, 18–20]. The average number of reported sinus infections, CRS-related antibiotics, and CRS-related oral corticosteroids per year were 3.53 ± 1.30, 2.44 ± 1.42, and 1.63 ± 1.13; respectively (Fig. 2a). Seven studies reported scores of the 22-item Sino- Nasal Outcome Test (SNOT-22) during active or inactive exacerbation in patients with CRS [6, 11, 13, 14, 18–20]. An average SNOT-22 score of 37.55 ± 21.06 was calculated during inactive exacerbation of CRS from six studies [6, 11, 14, 18–20]. Only two studies reported the changes in SNOT-22 score during AE in CRS [13, 14]. The average change of SNOT-22 scores during an exacerbation was 7.83 points higher as compared with routine visits.

**Table 2:** Quantifiable metrics of acute exacerbations in CRS

Author/year	Evaluation parameters of AE	Results	Conclusions	Level of evidence
Phillips, et al. (2017) [6]	Number of CRS-related antibiotics usage, sinus infections	SNOT-22 score is associated with the number of antibiotics; Antibiotic usage highly correlated with the number of reported sinus infections.	Sinusitis-related antibiotic usage, reflecting the frequency of acute CRS exacerbations, mediates the association between CRS symptomatology and QOL.	3a
Banoub, et al. (2018) [11]	Number of sinus infections, CRS-related antibiotics use, CRS-related oral corticosteroids use	The frequency of patient-reported sinus infections, CRS-related antibiotics courses and CRS-related oral corticosteroid courses was negatively associated with asthma control.	AE are negatively associated with the level of asthma control.	3a
Phillips, et al. (2019) [18]	Number of sinus infections, CRS-related antibiotics, CRS-related oral corticosteroids	AE positively associated with comorbid asthma, and SNOT-22.	A CRS exacerbation-prone phenotype characterized by high sinonasal disease burden with comorbid asthma.	3a
Sedaghat, et al. (2018) [19]	Number of sinus infections, CRS-related oral antibiotic, CRS-related oral corticosteroids	At least 1 course of antibiotics or oral corticosteroids in the last 3 months was the optimal threshold for detecting poorly controlled CRS.	Number of CRS-related antibiotic use, and CRS-related oral corticosteroid aid in the determination of global CRS control.	3a
Speth, et al. (2018) [20]	Number of sinus infections, CRS-related antibiotics, CRS-related steroid usage	SNOT-22 score ≥ 30 predicted at least 1 sinus infection, CRS-related antibiotics, or CRS-related oral corticosteroids in the past 3 months.	SNOT-22 score and AE were predictive of each other.	3a
Zemke, et al. (2019) [13]	SNOT-22 scores	SNOT-22 scores were 4.9 points higher during the AE	Changes of SNOT-22 indicate the AE.	3a
Diveka, et al. (2015) [14]	SNOT-22 scores	SNOT-22 scores were 10.75 higher during the AE	Changes of SNOT-22 indicate the AE.	3a

*CRS* Chronic rhinosinusitis, *AE* Acute exacerbation, *QOL* Quality of life, *SNOT-22* The 22-item Sino- Nasal Outcome Test

### Clinical risk factors for AE in CRS

We further summarized the clinical risk factors for the AE in CRS (Table 3 and Fig. 2b). Phillips et al. defined an exacerbation-prone CRS phenotype among patients who reported over 3 exacerbations in the preceding 12 months [18]. Comorbid asthma (adjusted OR = 3.68), elevated SNOT-22 scores (adjusted OR = 1.06) and nasal polyps (adjusted OR = 0.27) were significantly associated with an exacerbation-prone CRS phenotype (Fig. 2b). Furthermore, the SNOT-22 scores and AE of CRS were predictive of one other [20]. Rank et al. found that AE of CRS occurred in a distinct seasonal pattern where exacerbations were twice as likely to occur during winter months as compared with spring, summer, or fall [9]. Furthermore, Kuiper et al. found that body mass index, asthma, hay fever, sinus surgery history, and winter season consistently predicted acute exacerbations of nasal and sinus symptoms [15]. With respect to risk factor interactions, elevated baseline SNOT-22 were significantly and positively associated with antibiotic use in the past year [6]. Furthermore, asthma, the level of asthma control measured by Asthma Control Test (ACT) was negatively associated with the frequency of AE in CRS in the last 3 months, independent of CRS symptom severity [11]. For CRSwNP patients with asthma at the time of acute infectious episodes, positive bacterial cultures were obtained in 90.9% (10 out of 11) patients [21]. Kwah, et al. defined frequent exacerbations of CRS phenotype among patients who reported over 4 episodes over a 12-month period in which an antibiotic was prescribed for worsening sinus symptoms [22]. The authors found that frequent exacerbations of CRS were associated with comorbid asthma (adjusted OR = 2.61), allergic rhinitis (adjusted OR = 1.96), eosinophil count ≥150/μL (adjusted OR = 1.54), and autoimmune disease (adjusted OR = 1.68).

**Table 3:** Clinical risk factors for acute exacerbations in CRS

Author/year	Study design	Study participants	Definition of an acute exacerbation	Clinical risk factors	Results	Level of evidence
Rank, et al. (2010) [9]	Retrospective cohort study	CRS exacerbation	Systemic antibiotics, systemic corticosteroid, plans for a semi-urgent surgical intervention, emergency department or urgent care visit, or a hospitalization for CRS.	Winter months	Twice as likely to present for a CRS exacerbation in winter months compared with spring, summer, or fall.	4
Banoub, et al. (2018) [11]	Prospective	Asthmatic CRS	Sinus infections, CRS-related antibiotics use, CRS-related oral corticosteroids use.	Lower ACT scores (Poor asthma control).	AE are negatively associated with the level of asthma control in asthmatic CRS patients, independent of CRS symptom severity.	4
Kuiper, et al. (2018) [15]	Prospective	Acute exacerbations of NSS in patients with CRS	1.Self-reported medication use (antibiotics and oral corticosteroids) for worsened NSS; 2. duration (≥ 1 week) of worsened aggregate NSS; 3. duration (≥ 1 week) of worsened aggregate NSS and self-reported worsened purulence in the past 4 weeks.	Current long-term CRS status, high body mass index, asthma, hay fever, sinus surgery history, and winter season.	CRS status (current long-term, current recent, past, never), body mass index, asthma, hay fever, sinus surgery history, and winter season consistently predicted acute exacerbations of NSS.	4
Ikeda, et al. (2011) [21]	Prospective	CRSwNP with asthma undergoing ESS	Acute exacerbation of CRS defined by the presence of purulent sinonasal secretions in conjunction with sinus-related symptoms.	Bacterial infection	Positive culture was obtained in 10 out of 11 patients.	4
Kwah, et al. (2020) [22]	Retrospective cohort study	frequent exacerbations of CRS	worsening sinus symptoms and CRS-related antibiotics usage	Asthma, allergic rhinitis, eosinophil count ≥150/μL, and autoimmune disease	Frequent AECRS was characterized by a higher prevalence of asthma, allergic rhinitis, eosinophil count ≥150/μL, autoimmune disease.	4
Phillips, et al. (2018) [23]	Prospective	CRS exacerbation	CRS-related antibiotics usage, sinus infections.	High SNOT-22 scores.	SNOT-22 score is associated with the number of antibiotics which were highly correlated with the number of reported sinus infections.	4

*CRSwNP* Chronic rhinosinusitis with nasal polyps, *CRS* Chronic rhinosinusitis, *NSS* Nasal and sinus symptoms, *SNOT-22* The 22-item Sino- Nasal Outcome Test, *ACT* Asthma control test, *AE* Acute exacerbation, *ESS* Endoscopic sinus surgery

### In vivo immunologic characteristics of AE in CRS

**Fig. 3:**
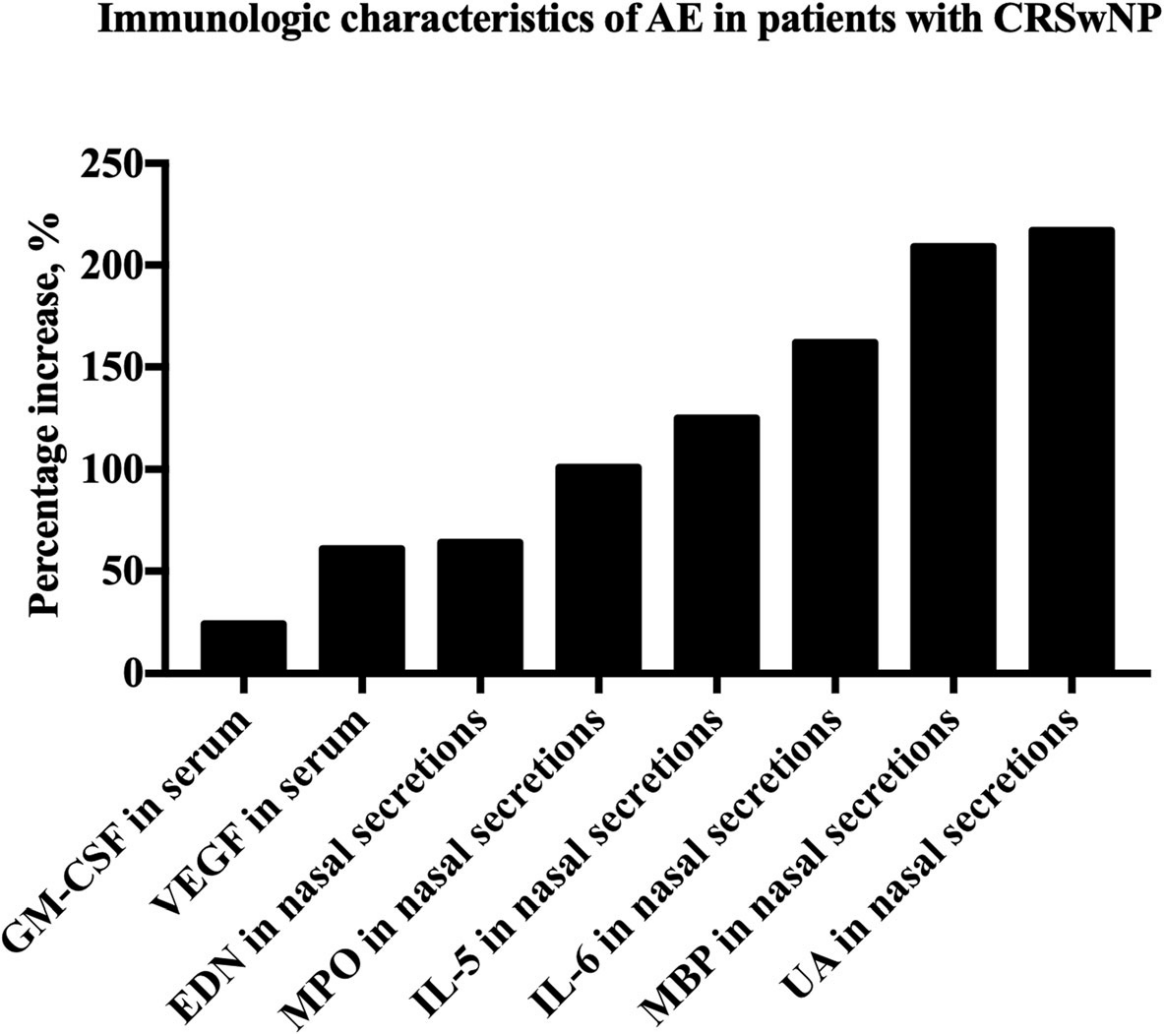
Immunologic characteristics of acute exacerbations in adult patients with CRS. AE, acute exacerbations; CRSwNP, chronic rhinosinusitis with nasal polyps; GM-CSF, granulocyte–macrophage colony-stimulating; VEGF, vascular endothelial growth factor; EDN, eosinophil-derived neurotoxin; MPO, myeloperoxidase; MBP, major basic protein; UA, uric acid

Immunologic characteristics of AE in CRSwNP in seven studies were summarized (Table 4 and Fig. 3). Local and systemic immune responses during AE in CRSwNP have been explored in two studies (Fig. 3) [14, 16]. Serum increases of 24 and 61% were observed in granulocyte–macrophage colony-stimulating factor (GM-CSF) and vascular endothelial growth factor (VEGF), respectively. With respect to nasal secretions, eosinophil-derived neurotoxin (EDN), myeloperoxidase (MPO), IL-5, IL-6, major basic protein (MBP), and uric acid (UA) were increased 64, 101, 125, 162, 209, and 217%, respectively. Five studies explored the biomarkers of AE in CRSwNP. A significant increase of mucus cystatin SA (CST2) and periostin (PST) was observed in patients with recurrent CRSwNP as compared with stable CRSwNP after surgery within the 2-year follow-up [24]. The percentage increase of mucus PST and CST2 were 9.69 and 246.42%, respectively. Another study by Ninomiya et al. showed that serum periostin higher than 115.5 ng/ml can predict the recurrence of CRSwNP [25]. Furthermore, significant expression of PST was highly associated with an eosinophilic CRSwNP [26], which is an intractable phenotype of CRSwNP. A study by Kato et al. also showed that significant higher level of Cystatin SN (CST1) mRNA in eosinophilc CRSwNP as compared with non-eosinophilc CRSwNP with a percentage increase of 983.33% [27], which was consistent with another study [28].

**Table 4:** Immunologic characteristics of acute exacerbations in CRSwNP

Author/year	Study groups	Immunologic characteristics	Level of evidence
Divekar, et al. (2015) [14]	CRSwNP (*n* = 9)	Significant increase of nasal IL-5、IL-6 and eosinophil major basic protein. Significant increase of serum VEGF and GM-CSF.	4
Rank, et al. (2013) [16]	CRSwNP (*n* = 10)	Significant increase of nasal IL-6, MBP, MPO, EDN and uric acid.	4
Mueller, et al. (2019) [24]	CRSwNP (*n* = 5)	Significant higher level of CST2 and periostin.	4
Ninomiya et al. (2018) [25]	ECRSwNP (*n* = 6)	Significant higher level of serum periostin.	4
Xu, et al. (2017) [26]	ECRSwNP (*n* = 30)	Significant higher level of serum periostin.	2c
Kato, et al. (2018) [27]	ECRSwNP (*n* = 51)	Significant higher level of CST1.	2c
Yan, et al. (2019) [28]	ECRSwNP (*n* = 192)	Significant higher level of serum periostin.	2c

*CRSwNP* Chronic rhinosinusitis with nasal polyps, *ECRSwNP* Eosinophilic chronic rhinosinusitis with nasal polyps, *VEGF* Vascular endothelial growth factor, *GM-CSF* Granulocyte–macrophage colony-stimulating, *MBP* Major basic protein, *MPO* Myeloperoxidase, *VEGF* Vascular endothelial growth factor, *EDN* Eosinophil-derived neurotoxin, *CST2* cystatin SA, *CST1* Cystatin SN

## Discussion

Defining acute exacerbations of CRS with quantifiable clinical and immunologic metrics has remained a challenge due to the complex etiology of acute exacerbation and the inconsistency in endpoint reporting. Current guidelines regarding treatment of AE in CRS continue to recommend blanket medical intervention including short-course antibiotic treatment [7, 8]. Thus, an improved method of clinically defining AE in CRS and its risk factors is critical to facilitate individualized treatment as well as more effective preventions aimed improving overall quality of life.

We first reviewed the current diagnostic criteria of AE in CRS and found certain discrepancies between studies. The definitions of AE in CRS rely largely on descriptive changes in sinonasal symptoms that occurred in an acute fluctuation pattern (Table 1) [7, 8, 13–15, 17, 29]. Consequently, we sought to identify more specific quantitative metrics which could be used to provide a common definition between studies. Patient-reported number of sinus infections and treatments-related metrics were available in several studies (Fig. 1) [6, 9, 10]. An exacerbation-prone CRS phenotype was defined as more than 3 times of exacerbations in the last 12 months [18]. Previous studies showed that asthma, elevated SNOT-22 scores, allergic rhinitis, eosinophil count of at least 150 cells per microliter, and autoimmune disease were positively associated with an exacerbation-prone CRS phenotype [18, 22]. However, the presence of nasal polyps was negatively associated an exacerbation-prone CRS phenotype [18]. This is similar to a previous study that found that CRSwNP are less likely to use antibiotics, which could serve as a proxy for an exacerbation, relative to other phenotypes of CRS patients [30]. SNOT-22 scores during active and inactive exacerbation were also characterized. A high average of SNOT-22 scores of 37.55 ± 21.06 was calculated from six studies indicating a high subjective burden of disease among exacerbation prone patients [6, 11, 14, 18–20]. In addition, average change in SNOT-22 score during an exacerbation was 7.83 points higher as compared with routine visits [13, 14]. The established minimal clinically important difference (MCID) for SNOT-22 is 9 to 12 based on two previous studies [23, 31]. While the SNOT-22 change during exacerbation appears slightly below this accepted MCID, this may be due to smaller sample size or the fact that the MCID was validated against therapeutic interventions, not spontaneous exacerbations of chronic disease.

We next summarized the clinical risk factors for AE in CRS. Winter season, hay fever, asthma, sinus surgery history, current long-term CRS status, and high body mass index were all independent risk factors which predicted AE in CRS [9, 15]. Furthermore, high baseline SNOT-22 scores and low ACT scores were highly associated with the frequency of AE in CRS in the preceding 3 months [6, 11]. Interestingly, a high positive bacterial culture rate of 90.9% was detected in patients during exacerbations [21]. These findings would facilitate preventive interventions on these risk factors to reduce exacerbation frequency.

Immunologic changes that occur during exacerbation offer a potential objective measure to confirm AE in CRS and perhaps even to predict it. The current literatures about the immunologic changes during exacerbation were all about patients with CRSwNP and studies about the immunologic changes of CRS without nasal polyps during exacerbation were lacking. From a systemic perspective, only GM-CSF and VEGF in patients with CRSwNP during exacerbation were significantly increased in serum samples when compared with controls [14]. Previous studies had shown that local production of both VEGF and GM-CSF in nasal polyps [32–34]. It is now understood that VEGF and GM-CSF play an important role in tissue remodeling and inflammatory responses. With respect to local nasal biomarkers, EDN, MPO, IL-5, IL-6, MBP, and UA within nasal secretions were all significantly increased when compared with controls [14, 22]. Previous studies have shown that EDN, IL-5 and MBP are involved in type 2 immune responses and eosinophilic inflammation [35]. MPO is a biomarker of the neutrophil activation and high levels of MPO represent increased neutrophilic inflammation [36]. Increased IL-6 response has been associated with onset of the viral upper respiratory tract infection in healthy individuals or virus-induced asthma exacerbations [37, 38]. High serum uric acid is an indicator of oxidative stress [39] and it has proved to be a marker of the severity of asthma exacerbations [40]. These studies thereby indirectly support the role of type 2 immune responses, neutrophilic inflammation and anti-viral activity in the pathogenesis of AE in CRSwNP. Interestingly, upregulation of these serum and nasal mucus biomarkers corresponded well with subjective patient-reported worsening of symptoms suggesting that these cytokines may indeed be a reliable measure of AE in CRSwNP.

Recent transcriptomic and proteomic studies exploring the pathogenesis of CRS have revealed a variety of upstream targets which appear to contribute to inflammation in CRSwNP [41, 42]. As these biomarkers may be non-invasively sampled in a prospective and serial manner, Mueller et al. demonstrated that mucus cystatin 2 and periostin levels were capable of predicting worsening SNOT-22 scores, a proxy for AE in CRSwNP, and even the need for revision surgery among patients with CRSwNP months in advance [24].

## Conclusions

AE in CRS has gained increasing attention due to its significant influence on patients’ quality of life and healthcare costs. The definition of acute exacerbation in patients with CRS to date has been generally descriptive. Our review of the literature suggests that AE in CRS is associated with an average of a 7.83-point decline in SNOT-22 and that patients with hay fever, asthma, sinus surgery history, current long-term CRS status, high body mass index, and during winter season as risk factors are at greatest risk of AE in CRS. Furthermore, AE in CRSwNP appears to be associated with significant increases in serum levels of GM-CSF and VEGF, as well as multiple nasal mucus cytokines including IL-5, IL-6, and MPO. Finally, recent studies suggest that upstream mucus biomarkers of Type 2 inflammation including Cystatin and Periostin may be used to predict an impending AE in CRSwNP. More studies are needed to build a consensus of the certain definition of AE in CRS which is based on the combination of the subjective and objective parameters.

## Data Availability

All data gathered for the systematic review was gathered from articles cited in the paper and listed in the reference section.

## Abbreviations

CRSwNP: Chronic rhinosinusitis with nasal polyps
AE: Acute exacerbations
CRS: Chronic rhinosinusitis
SNOT-22: The 22-item Sino-Nasal Outcome Test (SNOT-22)
OR: Odds ratio
PI: Percentage increase
PRISMA: Preferred Reporting Items for Systematic Reviews and Meta-Analysis
ACT: Asthma Control Test
EDN: Eosinophil-derived neurotoxin
MPO: Myeloperoxidase
MBP: Major basic protein
UA: Uric acid
CST2: Cystatin SA
CST1: Cystatin SN
PST: Periostin
MCID: Minimal clinically important difference
GM-CSF: Granulocyte macrophage colony-stimulating factor
VEGF: Vascular endothelial growth factor
